# A high-flexion design total knee prosthesis: a ten to twelve-year follow-up study

**DOI:** 10.1186/s13018-024-05082-3

**Published:** 2024-09-28

**Authors:** Man Soo Kim, Keun Young Choi, Jae Hyeong Hur, Yong In

**Affiliations:** Department of Orthopaedic Surgery, Seoul St. Mary’s Hospital, College of Medicine, The Catholic University of Korea, 222, Banpo-daero, Seocho-gu, Seoul, 06591 Republic of Korea

**Keywords:** High flexion, Total knee arthroplasty, Loosening, Range of motion, Clinical outcomes

## Abstract

**Background:**

The purpose of this study was to investigate the clinical and radiographic outcomes and to determine the survivorship of a high-flexion design total knee arthroplasty (TKA) prosthesis, the LOSPA knee system, over a follow-up period of 10–12 years.

**Methods:**

The study included 386 patients (503 TKAs) who were treated with TKA from 2011 to 2013 (follow-up period 10–12 years).The patients were assessed clinically using range of motion (ROM) of the knee, the Knee Society scoring system (KSS), and the Western Ontario and McMaster Universities Osteoarthritis Index (WOMAC). For radiographic analysis, the positions of femoral and tibial implants as α, β, γ, and δ angles, hip knee ankle (HKA) angle, and radiolucent lines were used. Kaplan–Meier survival analysis was performed.

**Results:**

Mean ROM improved significantly from the preoperative baseline of 117.3° to 126.5° at the final follow-up (*p* < 0.001). The mean KSS and WOMAC scores also both showed significant improvement after surgery (all *p* < 0.001). A non-progressive radiolucent line less than 2 mm was observed in 23 cases (4.7%). Nine patients underwent revision surgery on the knee during the follow-up period. Revision surgery was performed on four patients due to aseptic loosening, three patients due to infection, one patient due to ankylosis, and one patient due to instability. When the endpoint of survival was the entire surgical cases, the survival rate was 96.2%. The survival rate, with revision for any reason as the endpoint, was 97.2%, and 97.8% for aseptic causes.

**Conclusions:**

The LOSPA knee system, a high-flexion design total knee prosthesis, showed excellent long-term survivorship and improvements in clinical outcomes at 10- to 12-year follow-up.

## Background

Total knee arthroplasty (TKA) has revolutionized the treatment of end-stage knee osteoarthritis (OA), offering significant pain relief and improved function for patients [1–6]. The success of TKA is largely measured by the restoration of knee function and patient satisfaction, which are influenced by the degree of knee flexion achieved postoperatively [7, 8]. In daily activities across many cultures, high flexion beyond the standard 120° is needed, requiring prostheses that can support such a range of motion (ROM) [7]. Traditional TKA designs, while effective in alleviating pain and restoring basic function, often fall short in providing the high-flexion capability that many patients desire or require for their lifestyle and cultural practices [9, 10].

**Fig. 1:**
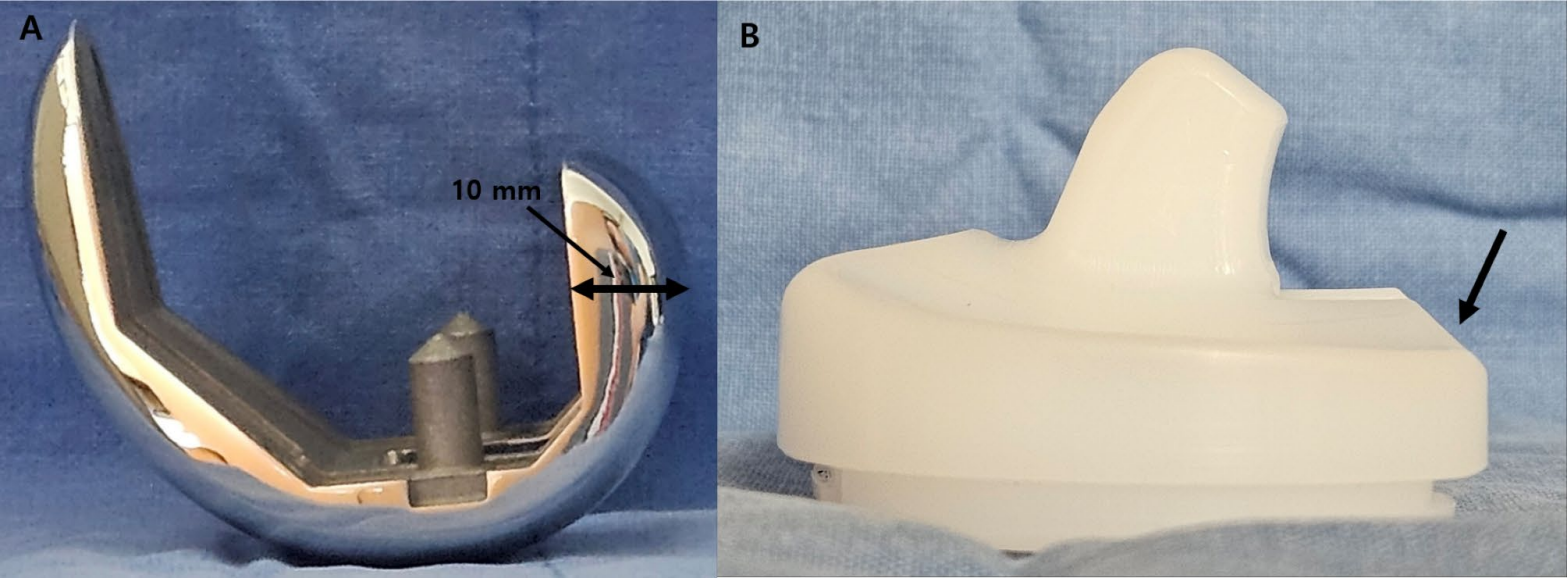
The LOSPA knee system (Corentec Inc., Seoul, Korea) necessitates the removal of additional bone from the posterior femoral condyle to accommodate a 10-mm posterior condyle with a larger posterior radius of the femoral component. The extension of the posterior condyle enhances the contact area at deep knee flexion angles, thus facilitating femoral rollback and increasing the range of flexion (**A**). The tibial insert is also designed to favor deep flexion. Its posterior surface is released, and the posterior edge is chamfered to prevent early bone-implant impingement (**B**)

The development of high-flexion knee prostheses, such as the LOSPA knee system (Corentec Inc., Seoul, Korea), represents a significant advancement in TKA design [11]. These prostheses are engineered to accommodate greater degrees of bending while maintaining implant longevity and stability [12]. This system necessitates the removal of additional bone from the posterior femoral condyle to accommodate a 10-mm posterior condyle with a larger posterior radius of the femoral component [13, 14]. The extension of the posterior condyle enhances the contact area at deep knee flexion angles, thus facilitating femoral rollback and increasing the range of flexion [13, 14]. Moreover, the femoral component features a more rounded contour and a deepened patellar groove, which aids in achieving deep flexion by reducing joint capsule overstuffing [12]. The tibial insert is also designed to favor deep flexion. Its posterior surface is released, and the posterior edge is chamfered to prevent early bone-implant impingement (Fig. 1) [12].

Since high-flexion design TKA prostheses were introduced, there has been a debate on the premature failure of these implants [15]. Some studies raised concerns about early loosening of the femoral component [16–19], while others reported no increased incidence of premature failure compared with traditional TKA implants [20–22]. Thus, the primary objective of this study was to conduct a comprehensive evaluation of the long-term outcomes associated with the use of the high-flexion concept LOSPA knee system in TKA. Given the paucity of long-term data on the performance and survivorship of high-flexion knee prostheses [11, 12], this study seeks to fill a critical gap in the literature. We assessed the clinical and radiographic outcomes and determined the survivorship of the LOSPA knee system over a follow-up period of 10–12 years. By providing a detailed analysis of these long-term outcomes, the study results offer valuable insights into the efficacy and durability of the LOSPA knee system, thereby informing clinical decision-making and future TKA implant design innovations.

## Patients and methods

**Fig. 2:**
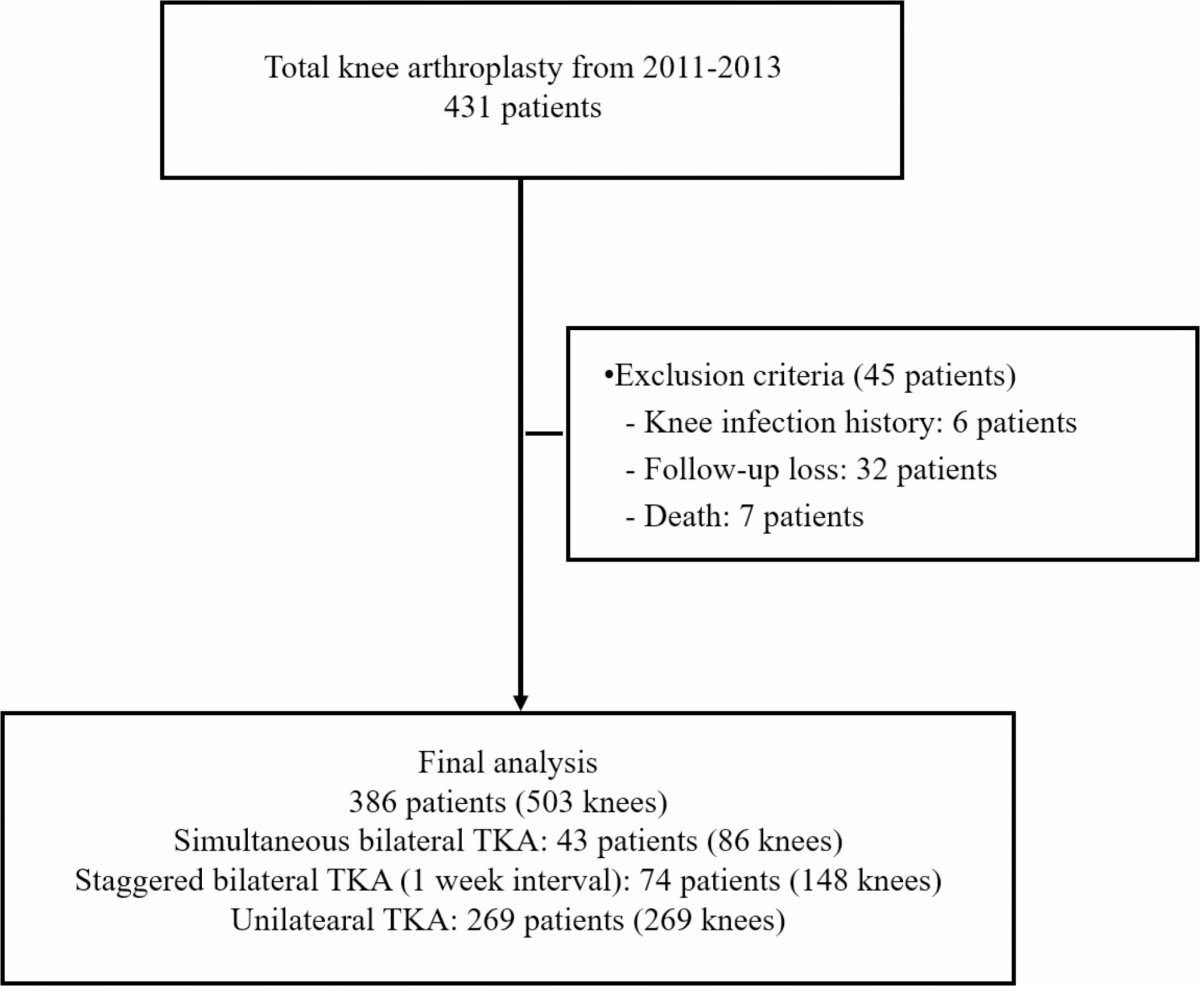
Flow chart

The Hospital Ethics Committee and Internal Review Board approved the present retrospective cohort study. This study was designed to evaluate the long-term outcomes and survivorship of TKA using the LOSPA high-flexion knee prosthesis. The study included patients who underwent TKA between June 2011 and December 2013, with a follow-up period of 10.3 ± 0.3 years postoperatively. Patients eligible for this study were those with primary TKA indications, such as severe knee OA rated grade 3–4 on the Kellgren–Lawrence scale [23], osteonecrosis, rheumatoid arthritis, and deformities like posttraumatic OA. Patients with a history of knee joint infection, patients with follow-up loss, and patients who died were excluded. Among the 431 eligible patients, 45 patients were excluded, including 6 patients with a history of knee joint infection, 32 patients with follow-up loss, and 7 patients who died. Ultimately, the study included 386 patients who underwent 503 TKAs (Fig. 2). Demographic data, including age, sex, body mass index (BMI), and preoperative diagnosis, were collected for all participants. The demographic details of the patients are provided in Table 1.

**Table 1:** Patient demographics

	LOSPA TKA group (*n* = 503 knees of 386 patients)
Gender (women : men)	353 : 33
Simultaneous bilateral : Staged (1 week interval) bilateral : Unilateral	86 : 148 :269
Age (years) *	69.8 ± 6.7 (42 to 86)
BMI (kg/m^2^) *	26.4 ± 3.5 (13.8 to 43.8)
Operation side (Right : Left)	249 : 254
Diagnosis(OA : RA : ON)	479 : 15 : 9
Follow up period (years)*	10.3 ± 0.3 (10.0 to 11.25)

*Values are mean and standard deviation with range in parentheses

BMI, body mass index; OA, osteoarthritis; RA, rheumatoid arthritis; ON, osteonecrosis

The senior surgeon (YI) at our facility performed all TKAs using the LOSPA PS knee system. The surgical approach was a subvastus approach carried out under general anesthesia with a tourniquet pressure of 300 mm Hg. Bone cuts and soft tissue releases were made to correct deformities, and gap balancing was assessed with spacer blocks and trial components. The LOSPA knee system was implanted using a modified measured resection technique, ensuring proper alignment and soft tissue balancing. Intraoperative measures were taken to achieve optimal positioning of the femoral and tibial components, with particular attention to rotational alignment. Most cases involved varus deformity, characterized by a femorotibial angle (FTA) greater than 0° on preoperative radiographs. For these cases, a deep medial collateral ligament (MCL) release was performed, followed by semimembranosus release or superficial MCL puncturing as needed to achieve a balanced mediolateral flexion-extension gaps difference within 2 mm [24]. Patellar resurfacing was done in 347 cases (69.0%), and the femur and tibia components were fixed using bone cement (Palacos R + G, Heraeus Medical, Wehrheim, Germany). Postoperative care followed a standardized protocol, including antibiotic prophylaxis, venous thromboembolism prevention, and a rehabilitation program aimed at early mobilization and range of motion exercises. Quadriceps strengthening exercises were started immediately after surgery. On the first day after surgery, patients began walking using a walker and began active ROM exercises under the guidance of medical staff.

Clinical evaluations were conducted before and after surgery to assess the Knee Society Score (KSS) [17], Western Ontario and McMaster Universities Osteoarthritis Index (WOMAC) score [25], and ROM. The KSS has been widely validated and is considered one of the most reliable tools for assessing outcomes after TKAs [26]. Its comprehensive nature allows for the detailed evaluation of both the mechanical function of the knee and the patient’s ability to perform daily activities [26]. The WOMAC index is considered a gold standard for measuring the effectiveness of treatments for OA, including surgical interventions like TKA [25]. Its specificity to OA symptoms and impact on quality of life, along with its extensive validation in diverse patient populations, underscores its utility as an outcome measure tool in both clinical and research settings [25]. Each item on the WOMAC is scored using a Likert scale, typically ranging from 0 (no symptoms) to 4 (extreme symptoms), with a total score ranging from 0 to 96 [25]. Higher scores indicate worse pain, stiffness, and functional limitations [25]. Active ROM of the knee was measured with a standard 60-cm goniometer in the supine position by one of the authors with no knowledge of the patient’s name.

Preoperative and postoperative radiological evaluations included standing anteroposterior (AP), lateral, merchant, and long-standing radiographs from the hip to the ankle. The alignment of the lower limb was determined using the femorotibial angle (FTA) and hip-knee-ankle (HKA) angle based on the long-standing radiographs from the hip to ankle. The FTA is the angle between the anatomical axes of the femur and tibia. The HKA angle is the angle between the mechanical axes of the femur and tibia [11, 12]. Radiographic evaluations were conducted to assess the positioning and integrity of the femoral and tibial components. The analysis focused on coronal angle of the femoral component (α), coronal angle of the tibial component (β), flexion angle of the femoral component (γ), and the tibial slope angle (δ) angles to ensure positioning of the implants [27]. AP and lateral radiographs were evaluated for radiolucent lines (RLLs) at the bone–cement interfaces using the Knee Society Radiographic Evaluation System [28]. RLLs are defined as clear gaps between the bone and cement [11, 12]. Radiographic findings of lines wider than 2 mm, osteolysis, and loosening of implants were noted as radiological abnormalities [29]. The presence and progression of radiolucent lines were also evaluated as indicators of implant loosening or failure. Radiographic measurements were independently taken by two orthopedic surgeons who were not involved in the surgical procedures and were blinded to the patient information. The average measurements obtained by the two readers were used. The reproducibility of the measurements was high, as indicated by an intraclass correlation coefficient of 0.85 (*p* < 0.001).

The endpoint of survival was set in three ways. First, entire operation cases were used to calculate survival rate. Second, only revision cases including infection were used. Third, revision cases not including infection were used. Additionally, surgical and medical complications after surgery were recorded.

### Statistical analysis

Continuous variables, such as ROM, KSS, and WOMAC scores, were expressed as mean ± standard deviation and compared using the paired *t-*test or Wilcoxon signed-rank test as appropriate. Kaplan–Meier survival analysis was used to estimate the survivorship of the LOSPA knee system, with operation for any reason and revision operation for any reason and for aseptic loosening as endpoints. Data were analyzed using SPSS 21 software (SPSS Inc., Chicago, IL, USA). A *p*-value of less than 0.05 was considered statistically significant.

## Results

Patient-reported outcome measures exhibited substantial improvements. The average KSS showed significant improvement, moving from 105.1 (20 to 185) before surgery to 168.9 (100 to 200) at the last check-up (*p* < 0.001). The WOMAC score significantly improved from 57.1 (23 to 88) preoperatively to 24.5 (4 to 63) last check-up after surgery (*p* < 0.001) (Table 2).

**Table 2:** Preoperative and postoperative clinical scores

	Preoperative	Preoperative (Last Follow-up)	*P* value
KSS	105.1 ± 27.8 (20 to 185)	168.9 ± 18.7 (100 to 200)	< 0.001
Pain	25.0 ± 9.1 (0 to 50)	46.0 ± 6.1 (10 to 50)	< 0.001
Function	80.2 ± 24.1 (10 to 140)	122.9 ± 16.5 (50 to 150)	< 0.001
WOMAC	57.1 ± 13.1 (23 to 88)	24.5 ± 9.9 (4 to 63)	< 0.001
Pain	11.3 ± 3.2 (3 to 21)	2.2 ± 2.8 (0 to 15)	< 0.001
Stiffness	3.9 ± 1.9 (0 to 8)	1.8 ± 1.8 (0 to 8)	< 0.001
Function	41.9 ± 10.4 (11 to 71)	20.6 ± 8.2 (3 to 60)	< 0.001

*Values are mean and standard deviation with range in parentheses

KSS, Knee Society score; WOMAC, Western Ontario and McMaster Universities score

The mean ROM showed a significant improvement from the preoperative baseline of 117.1° (40 to 140°) to 128.2° (80 to 144°) at the final follow-up (*p* < 0.001). The mean preoperative HKA showed a varus of 8.0° (valgus 7.0 to varus 26.2°), shifting to a postoperative varus of 1.1° (valgus 5.3 to varus 9.7°), indicating a significant correction (*p* < 0.001) (Table 3). The coronal angle of the femoral component (α) was 96.3° (85.8 to 102.2°), the coronal angle of the tibial component (β) was 89.1° (81.6 to 97.8°), the flexion angle of the femoral component (γ) was 5.0° (0 to 14.3), and the tibial slope angle (δ) was 86.3 (79.2 to 96.0°) postoperatively. Non-progressive RLLs were found in 23 knees (4.6%): 10 in the medial tibial condyle (zones 1 and 2), 12 in the anterior femoral condyle (zones 1 and 2), and 5 in the posterior femoral condyle (zones 3 and 4). There were four knees in which radiolucent lines were found in both the anterior and posterior femoral condyle.

**Table 3:** Preoperative and postoperative alignment and range of motion

	Preoperative	Postoperative (Last Follow-up)	*P* value
Knee alignment			
HKA(°)	Varus 8.0 ± 5.3 (Valgus 7.0 to varus 26.2)	Varus 1.1 ± 2.5 (Valgus 5.3 to varus 9.7)	< 0.001
FTA(°)	Varus 3.6 ± 5.4 (Valgus 12.3 to varus 19.8)	Valgus 5.3 ± 2.5 (Valgus 10.9 to varus 4.7)	< 0.001
ROM (°)	117.1 ± 12.2 (40 to 140)	128.2 ± 6.5 (80 to 144)	< 0.001
Flexion contracture (°)	6.4 ± 6.0 (0 to 30)	0.2 ± 1.2 (0 to 10)	< 0.001
Maximum flexion (°)	123.5 ± 10.2 (60 to 140)	128.4 ± 6.0 (90–144)	< 0.001

*Values are mean and standard deviation with range in parentheses

HKA, Hip knee ankle angle; FTA, femoro-tibial angle; ROM, range of motion

**Fig. 3:**
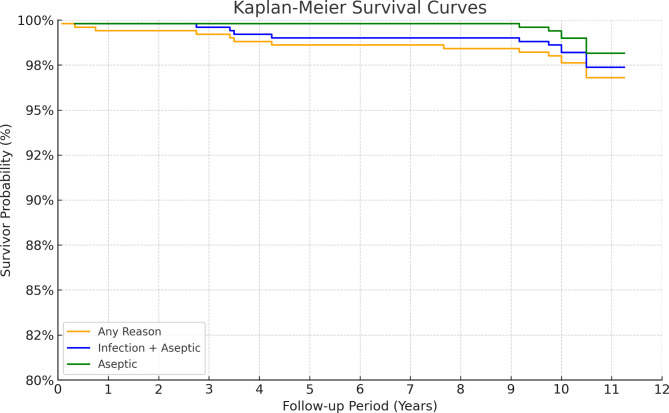
Kaplan–Meier survival analyses for the endpoints aseptic loosening (98.2%), all revisions including infection (97.4%) and all reoperations (including revision) (96.8%)

When the endpoint of survival was the entire surgical cases, the survival rate was 96.8%. The survival rate, with revision for any reason as the endpoint, was 97.4 and 98.2% for aseptic causes (Fig. 3). Eight cases (1.6%) underwent revision surgery during the follow-up period. Among these, three patients underwent revision surgery due to deep infection, three patients due to aseptic loosening of femoral or tibial component, one patient due to recurvatum instability, and one patient due to ankylosis. Additionally, one patient experienced deep infections that were managed successfully with synovectomy and polyethylene change, and three patients required surgery for periprosthetic fractures of the femur and patella (Tables 4 and 5). One patient had a periprosthetic fracture treated with a cast. Cerebral infarction, acute kidney injury, and ischemic hepatitis occurred in two patients each (Table 5).

**Table 4:** Details of patients who underwent any operation

Case Number	Age	Sex	Indication	Side	Mode of failure	Management	Survival (months)
1	76	Female	OA	Right	Aseptic loosening	Revision	120
2	65	Female	OA	Right	Flexion contracture	Revision of femoral component	4
3	60	Female	OA	Left	Tibial component debonding	Revision of Tibial component	117
4	59	Female	OA	Left	Recurvatum	Revision	126
5	68	Female	OA	Left	Infection	Two-stage revision	41
6	55	Female	OA	Right	Debonding of femoral component	Revision of femoral component	110
7	78	Female	OA	Right	Patella fracture	Open reduction and internal fixation	1
8	75	Female	OA	Right	Infection	Two-stage revision	51
9	68	Female	OA	Right	Infection	*DAIR* debridement and implant retention	33
10	68	Female	OA	Left	Distal femur periprosthetic fracture	Open reduction and internal fixation	92
11	84	Female	OA	Right	Infection	Two-stage revision	42
12	71	Female	OA	Left	Distal femur periprosthetic fracture	Open reduction and internal fixation	9

OA, osteoarthritis

**Table 5:** Complications

Complications	Number (%)
Aseptic loosening	4 (0.8%)
	4 cases : Revision operation
Infection	5 (1.0%)
	3 cases: Infection control & Revision operation
	1 case: Open debridement & Polyethylene change
	1 case: Intravenous antibiotics treatment
Recurvatum instability	1 (0.2%): Revision operation
	1 case: Revision operation
Ankylosis (Stiffness)	2 (0.4%)
	1 case: Revision operation
	1 case: Manipulation (Brisement)
Periprosthetic fracture	5 (1.0%)
	4 cases: Open reduction and internal fixation
	1 case: Cast treatment
Cerebral infarction	2 (0.4%)
DVT & PTE	2 (0.4%)
Arrhythmia	2 (0.4%)
Wound dehiscence	3 (0.6%)
Derilium	2 (0.4%)
Acute Kidney Injury	2 (0.4%)
Postoperative ischemic hepatitis	2 (0.4%)
Urinary difficulty	2 (0.4%)
Cellulitis	1 (0.2%)

DVT, deep vein thrombosis; PTE, pulmonary thromboembolism

## Discussion

This study presents a comprehensive evaluation of the long-term outcomes and survivorship of TKA using the LOSPA high-flexion knee system over a follow-up period of 10–12 years. The findings revealed a significant improvement in the ROM, with patients achieving an average of 128.2° at the last follow-up, indicating the system’s effectiveness in facilitating high-flexion postoperatively. Both the KSS and the WOMAC scores showed marked improvements, reflecting enhanced knee function, reduced pain, and improved quality of life for the patients. Radiographic analysis revealed minimal occurrences of non-progressive radiolucent lines, suggesting stable implant fixation and alignment over time. The survival rates of 98.2% for aseptic causes and 96.8% for entire surgical cases with any reason underscore the durability and reliability of the LOSPA knee system in the long term. A significant finding was that the high-flexion LOSPA prosthesis did not exhibit early aseptic loosening.

Postoperative ROM is crucial for patient satisfaction following TKA, especially in Asian populations where daily activities often require deep knee flexion exceeding 125° [30]. To meet this need, prosthesis designs have been enhanced to facilitate greater knee flexion post-surgery [31]. Numerous studies have investigated ROM outcomes following high flexion TKA, but the results have varied [31–37]. Some studies comparing high flexion TKA to conventional TKA reported no significant differences in ROM [33, 34, 36], while others noted improved ROM with high flexion TKA [31, 32, 35, 37]. In our study, the postoperative ROM improved from a preoperative mean of 117.1° to 128.2°, and further flexion improved from 123.5° preoperatively to 128.4° postoperatively. Although these improvements indicate enhanced ROM and further flexion angles post-surgery, they fall short of the angles intended with high flexion TKA designs. Comparative analysis with another high-flexion prosthesis revealed that the mean postoperative flexion angle using the LOSPA system was 126.7°, which aligns with our findings [11]. Despite the theoretical capability of high flexion TKA designs to allow flexion angles up to 140°, achieving such degrees of flexion in practice remains challenging [12]. This discrepancy suggests that while high flexion TKA has potential advantages, translating these theoretical benefits into actual clinical outcomes remains complex and warrants further investigation [11, 12, 33, 34, 36].

There is a debate regarding the potential for high-flexion TKA to pose a higher risk of aseptic loosening [18, 38]. Some studies indicate that high flexion generates larger net quadriceps moments and net posterior forces compared with those associated with routine activities, potentially leading to pathological changes in the knee joint [39]. Additionally, high contact stress on the post-cam mechanism during high flexion has been proposed as a contributing factor [40]. Shiramizu et al. suggested that high-flexion TKAs could result in almost point contact at extreme ROM, increasing polyethylene wear and osteolysis [41]. Cho et al. reported that 13.8% of 218 high-flexion TKA cases exhibited progressive RLL around the femoral component after an average follow-up of 51 months [38]. Han et al. found a 38% rate of aseptic loosening in a study of 72 high-flexion TKA cases over a mean follow-up of 34 months [18]. Another study comparing 851 high-flexion TKA cases to 1,512 conventional TKA cases reported aseptic loosening in 4.9% of high-flexion TKA patients and 0.6% of conventional TKA patients over an average follow-up of about 70 months [33]. Conversely, other reports suggest that high-flexion TKA can provide a wider contact area and reduce contact stress during high-flexion movements [14, 42]. Several studies from Asia have reported favorable long-term outcomes without early aseptic loosening in high-flexion TKA patients [35, 43, 44]. Kim et al. reported a 10-year survivorship rate of 99% for high-flexion TKA, with no cases of aseptic loosening [43]. In our study, aseptic loosening was observed in three cases (0.6%), with femoral component loosening in two cases (0.4%). Over a 10-year follow-up period, revision due to implant loosening was required in 3 out of 504 cases, indicating over 98% survivorship, comparable to conventional posterior-stabilized (PS) TKA [45]. Overall, the long-term follow-up results for the LOSPA TKA were stable.

In general, modern high-flexion TKA prostheses feature increased posterior condylar metal thickness and/or posteriorly beveled tibial inserts [17]. The LOSPA high-flexion system, classified as a high-flexion TKA, extends the posterior condyle of the femoral component to increase the articular contact area in high flexion, necessitating an additional 2-mm bone resection from the posterior femoral condyles [11, 12]. In conventional TKA, the load is shared between the femoral component and the posterior condylar bone during high flexion [43]. However, in high-flexion TKA, the extent of load sharing in high flexion is reduced, which can result in greater shear forces in the femoral component [39, 40]. Cho et al. reported that progressive RLLs were observed all around the femoral components, not around the tibial component [38]. Nevertheless, in our study, the rate of RLL was only 4.6%. Whether this factor directly contributes to increased mechanical loading of the femoral component during high flexion remains unclear [43]. However, in our study, femoral component loosening was observed in only two cases over a 10-year follow-up period, none of which were progressive.

In the long-term follow-up of TKA, the most important outcome is implant survivorship [45]. Meta-analysis studies reported a 15-year survivorship rate of 96.3% for TKA, while the combined results from the Australian and Finnish registries report a rate of 93% [45]. Radetzki et al. reported that 3 out of 17 (18%) high-flexion TKAs required revision at an average follow-up of 10 years, leading the researchers to suggest that the use of high-flexion TKA systems should be reconsidered [46]. Conversely, Crawford et al. found that the survival rate of high-flexion TKAs was 96.4% at 10 years for aseptic loosening and 95.5% for all causes [34]. Similarly, Kim et al. reported a 20-year survival rate of 94.8% for the high-flexion TKA group [43]. In Asian countries, where high-flexion activities are frequently required, there have been numerous reports on high-flexion TKA [7]. In our study, over 10-year follow-up results showed a survivorship of over 96% for all causes or over 98% for aseptic causes.

This study has several limitations. First, more than 90% of patients included in this study were women. Most patients with end-stage knee OA who undergo TKA in Asian countries are women [47, 48]. Second, no patients in this study were morbidly obese and the preoperative range of knee motion was generally good. These factors may limit the generalizability of our findings to other patient groups or practice settings [11, 12]. Third, it was designed as a case series rather than a comparative investigation, lacking a control group for a direct comparison of ROM and both radiographic and clinical outcomes [11]. Fourth, the study focused solely on one type of high flexion PS TKA prosthesis. Therefore, the findings may not be applicable to other types of high-flexion TKA designs or to cruciate-retaining designs. Finally, the study’s focus on radiographic and clinical outcomes may overlook other factors influencing TKA success, such as patient activity levels and comorbidities. The association between postoperative lifestyle and loosening in patients who underwent TKA was not evaluated [27]. To understand the factors associated with loosening of the femoral component in high-flexion TKA, it is necessary to assess whether patients continue activities requiring high flexion even after TKA [27].

Future research should aim to address the limitations of this study by incorporating prospective designs, control groups, and longer follow-up periods. Comparative studies evaluating the LOSPA system against other high-flexion and conventional TKA designs could provide deeper insights into the relative performance of the LOSPA system [12, 14, 36, 43]. Investigating the impact of patient-specific factors on outcomes could also enhance the understanding of the LOSPA system’s applicability to diverse patient populations [12, 14, 36, 43].

## Conclusion

In conclusion, the LOSPA high-flexion knee system has demonstrated excellent long-term survivorship and significant improvements in clinical outcomes, making it a viable option for patients undergoing TKA, particularly those with high demands for postoperative knee flexion. These findings contribute valuable insights into the field of orthopedic surgery, supporting the continued advancement and refinement of TKA designs to meet the evolving needs of patients.

## Data Availability

The datasets used and analyzed during the current study available are from the corresponding author upon reasonable request.

## Abbreviations

TKA: Total Knee Arthroplasty
ROM: Range of Motion
KSS: Knee Society Scoring System
WOMAC: Western Ontario and McMaster Universities Osteoarthritis Index
HKA: Hip Knee Ankle
OA: Osteoarthritis
FTA: Femorotibial Angle
MCL: Medial Collateral Ligament
AP: Anteroposterior
RLLs: Radiolucent Lines
PS: Posterior Stabilized
